# The Interactive Effects of Cytoskeleton Disruption and Mitochondria Dysfunction Lead to Reproductive Toxicity Induced by Microcystin-LR

**DOI:** 10.1371/journal.pone.0053949

**Published:** 2013-01-16

**Authors:** Liang Chen, Xuezhen Zhang, Wenshan Zhou, Qin Qiao, Hualei Liang, Guangyu Li, Jianghua Wang, Fei Cai

**Affiliations:** 1 Fisheries College, Huazhong Agricultural University, Wuhan, China; 2 Key Laboratory of Freshwater Animal Breeding, Ministry of Agriculture, Wuhan, China; 3 Department of pharmacology, Medical College, Xianning University, Xianning, China; Centro de Investigación en Medicina Aplicada (CIMA), Spain

## Abstract

The worldwide occurrence of cyanobacterial blooms evokes profound concerns. The presence of microcystins (MCs) in waters and aquatic food increases the risk to human health. Some recent studies have suggested that the gonad is the second most important target organ of MCs, however, the potential toxicity mechanisms are still unclear. For a better understanding of reproductive toxicity of MCs on animals, we conducted the present experimental investigation. Male rats were intraperitoneally injected with MC-LR for 50 d with the doses of 1 and 10 µg/kg body weight per day. After prolonged exposure to MC-LR, the testes index significantly decreased in 10 µg/kg group. Light microscope observation indicated that the space between the seminiferous tubules was increased. Ultrastructural observation showed some histopathological characteristics, including cytoplasmic shrinkage, cell membrane blebbing, swollen mitochondria and deformed nucleus. Using Q-PCR methods, the transcriptional levels of some cytoskeletal and mitochondrial genes were determined. MC-LR exposure affected the homeostasis of the expression of cytoskeletal genes, causing possible dysfunction of cytoskeleton assembly. In MC-LR treatments, all the 8 mitochondrial genes related with oxidative phosphorylation (OXPHOS) significantly increased. The reactive oxygen species (ROS) level significantly increased in 10 µg/kg group. The mitochondria swelling and DNA damage were also determined in 10 µg/kg group. Hormone levels of testis significantly changed. The present study verified that both cytoskeleton disruption possibly due to cytoskeletal reorganization or depolymerization and mitochondria dysfunction interact with each other through inducing of reactive oxygen species and oxidative phosphorylation, and jointly result in testis impairment after exposure to MC-LR.

## Introduction

Cyanobacterial blooms and the associated cyanotoxins contamination are being increasingly reported worldwide. These toxins can be accumulated in aquatic organisms and transferred to higher trophic levels, representing a health hazard to animals and humans [1], [2]. Among all the cyanotoxins, microcystins (MCs) are the most frequently studied because of their wide distribution and high toxicity. Up to now, more than 80 different structural variants of MCs have been identified [2], among which microcystin-LR (MC-LR) is the most common and potent variant, followed by microcystin-RR (MC-RR) and microcystin-YR (MC-YR) [3]. Microcystins are hepatoxic toxicants, and are known to be highly potent and specific inhibitors of eukaryotic protein serine/threonine phosphatases 1 and 2A (PP1 and PP2A) [4], which in turn causes hyperphosphorylation of key control proteins that regulate tumor promotion or apoptosis [5].

Gonads have been regarded as the second important target organs of MCs [6]. Recent studies have verified that MCs accumulated in testis, and exerted toxic effects on reproductive system [7], [8], [9], [10], [11]. However, the underlying mechanisms of reproductive toxicity of MCs are still unclear. Many studies have investigated reproductive toxicity of MCs on male mammals. MCs induce morphological damages [7], [8], [12], and result in significant decrease of sperm quality [7], [12], [13], and also cause decline of some serum hormones, including testosterone, follicular stimulating hormone (FSH) and luteinizing hormone (LH) levels [7]. Most of previous studies are willing to attribute the testes damage to apoptosis and oxidative stress caused by MCs [7], [9], [14], [15], [16], [17].

Our previous proteomic investigations indicate that 20 of 40 significantly changed proteins are cytoskeleton assembly proteins in zebrafish embryos treated with MC-LR [18]. The cytoskeleton, consisting of three major elements: microfilaments (MFs), microtubules (MTs) and intermediate filaments (IFs), is a network structure composed of many kinds of structural and contractile proteins and plays an important role in cellular structural stability, intracellular transport and endocytosis [19]. Cytoskeletal alterations, including reorientation and depolymerization associated with MCs exposure have been presented in a number of studies [20], [21], [22]. Several studies have observed hyperphosphorylation of cytoskeletal proteins induced by MC-LR [23], [24], [25], which in turn leads to disruption of many cellular processes, alteration, breakdown and reorganization of the cytoskeleton [19], [26], [27], [28], [29], loss of intercellular contacts [25], [26], [30], and consequently disruption of cellular architecture. Some remarkably altered proteins in zebrafish treated with MC-RR are involved with cytoskeleton assembly [10]. However, as to the impairment of mammal reproductive system caused by MCs, very few studies have focused on cytoskeleton disruption.

Mitochondria are known to be vulnerable targets of various toxins because of their important role in maintaining cellular structures and functions [31]. Several studies have reported MC induced ultrastructural damage of mitochondria in liver [32], [33], kidney [34], heart [33] and testis [8], [10]. MCs also result in the onset of mitochondrial permeability transition (MPT) and loss of mitochondrial membrane potential (MMP) [35], [36], [37], [38]. However, the mechanism of testes mitochondria damage caused by MCs has rarely been reported.

Based on the above analysis, we advanced our hypothesis that cytoskeleton disruption and mitochondrial dysfunction of testis should be responsible for reproductive toxicity of MCs. In the present study, rats were administered a consecutive intraperitoneal injection of MC-LR for 50 d at doses of 1 and 10 µg/kg body weight per day. We verified our hypothesis mainly from the following aspects: 1) characterizing MC-induced morphological damages in testis and mitochondrial swelling and DNA damage; 2) determining the transcriptional levels of cytoskeleton and mitochondrial genes using Q-PCR methods; 3) measuring the formation of reactive oxygen species (ROS); 4) evaluating testosterone levels of testicular tissue. Our research will improve the understanding at molecular level of reproductive toxicity of MCs on mammals from a fresh point.

## Materials and Methods

### Chemicals

Purified MC-LR (purity ≥98%) was purchased from Alexis Biochemicals (Lausen, Switzerland) and confirmed using high performance liquid chromatography (HPLC, LC-10A, Shimadzu Corporation, Nakagyo-ku, Kyoto, Japan) following the method described by [39]. The chemical was dissolved in physiological saline solution (0.9% NaCl) as a vehicle for injection. Testosterone, luteinizing hormone (LH) and follicle stimulating hormone (FSH) Radioimmunoassay kits were purchased from Beijing North Institute of Biological Technology (Beijing, China). All of the other reagents utilized in this study were of analytical or higher grades.

### Animals and Ethics Statement

Male Wistar rats weighing 180–200 g were supplied by Hubei Laboratory Animal Research Center of Hubei Province, China. Rats were kept in stainless steel cages with accessible food and water ad libitum under constant conditions of 12 h light/dark cycle. No rats died during the 50 d exposure. This study was approved by the Institutional Animal Care and Use Committee (IACUC) of Huazhong Agricultural University, Wuhan, Hubei Province of China, and all efforts were made to minimize animal suffering to reduce the number of animals used.

### Toxin Exposure

The rats were randomly divided into three groups. The MC-LR treatment rats received intraperitoneal injection (i.p.) of MC-LR (1 µg/kg or 10 µg/kg per day, n = 10 per group) for 50 d, and were expressed as low dose group and high dose group, respectively. The control group (n = 10) was injected with the same volume of 0.9% saline solution.

### Sampling and Weights of Testes

After 12 h of the last intraperitoneal injection, the rats were weighed, and then sacrificed by neck vertebra luxation. The testes from each rat were excised free of surrounding connective tissues and weighed separately. Testis index was calculated by the formula: (testes weight/body weight) ×100%.

### Light Microscopy

For qualitative analyses of testicular histology, testes of control or MC-treated rat were fixed in Bouin’ s solution for 24 h, then washed in 70% alcohol to remove excess picric acid. After dehydration in a graded series of ethanol and clearing in xylol, samples were embedded in paraffin wax and blocks were prepared and sectioned on a microtome. The 5 µm thick testicular sections collected from each testis were stained with hematoxylin and eosin (H&E), and dehydrated in a graded series of alcohol, cleared in xylol, and mounted in neutral gum. Histological analysis was performed using light microscopy.

### Transmission Electron Microscopy

For transmission electron microscopic study, specimens of the gonad tissues were diced into 1 mm^3^, prefixed in 2.5% glutaraldehyde solution, followed by three 15 min rinses with 0.1 M phosphate buffer (pH 7.4). Post-fixation was in cold 1% aqueous osmium tetroxide for 1 h. After rinsing with phosphate buffer again, the specimens were dehydrated in a graded ethanol series of 50–100% and then embedded in Epon 812. Ultrathin sections were sliced with glass knives on a LKB-V ultramicrotome (Nova, Sweden), stained with uranyl acetate and lead citrate and examined under a HITACHI, H-600 electron microscope.

### Mitochondria Extraction

Mitochondria were isolated from testis of rats by standard differential centrifugation [38]. Testes excised from rat were homogenized in an ice-cold isolation medium containing 0.25 M sucrose, 20 mM HEPES, 1 mM ethylene-diaminetetraacetic (EDTA), 0.2% (w/v) defatted bovine serum albumin, pH 7.4, and homogenized (10 mL/g) using a homogenizer. Nuclei and unbroken cells were separated from the homogenate by centrifugation at 700 × g for 10 min at 4°C, and the mitochondria were separated from the supernatant by centrifugation at 10, 000 × g for 10 min at 4°C. The mitochondrial pellet was washed twice and resuspended in a medium containing sucrose 0.25 M, 20 mM HEPES, pH 7.4 and suspended at 15–30 mg of protein/mL. The protein concentration of mitochondrial suspension was measured by the Bradford method [40], using bovine serum albumin (BSA) as a standard.

### Mitochondrial Swelling

Mitochondrial swelling was assayed from the decrease in the absorbance at 520 nm [41]. Isolated mitochondria (0.1 mg protein/mL) from testis were suspended in swelling buffer [that contained (in mM) 120 KCl, 10 Tris-HCl (pH 7.4), 20 MOPS, and 5 KH_2_PO_4_]. The absorbance change was measured using a spectrophotometer.

### Mitochondrial DNA Isolation, Electrophoresis

Mitochondrial DNA from samples of control and microcystin-treated testis was isolated according to the method of Palva and Palva [42]. Electrophoresis was carried out for 0.5 h in 1% agarose gel. DL 2000 DNA marker was served as molecular size standard. MtDNA was stained with GelGreen.

### Hormone Assay

The gonad tissues were homogenized in 1.5 mL 70% (v/v) methanol (4°C) by a homogenizer according to Lun et al. [43]. Homogenates were centrifuged at 2500 × g (4°C) for 25 min and the supernatants were collected. The levels of testosterone, FSH and LH were measured by radioimmunoassay (RIA), according to the manufacture’s instructions. Because commercially available kits are for human, the comparative levels of testosterone, FSH and LH could be detected, but the absolute levels not.

### Determination of ROS Production

The gonad tissues were homogenized in physiological saline (0.9% NaCl, 4°C) by a homogenizer. Homogenates were centrifuged at 10000 × g (4°C) for 15 min and the supernatants were collected and used to measure levels of ROS using ELISA method. Briefly, ninety-six-well plate was coated with 50 µL of 5 µg/mL mice anti-rat ROS (Rapidbio, USA) per well in PBS overnight. The plates were then washed three times with PBS and blocked with PBS for 2 h at 37°C. Totally, 50 µL of standard control and samples were added into each well, and then horseradish peroxidase (HRP)-conjugate antibody against ROS was added. After 1 h incubation at 37°C, the plate was washed with PBS. Following five washes, color was developed by adding 3, 3′, 5, 5′-tetramethyl benzidine (TMB) substrate. Finally, 50 µL of the stop solution were added and the optical density was measured at 450 nm using a microplate reader. ROS concentration was assessed in triplicates. The protein concentration of the supernatants was determined by the Bradford method [40], using BSA as a standard protein.

### Total RNA Isolation and Reverse Transcription

Total RNA was isolated from 50 to 100 mg sections of testis using Trizol reagent (Invitrogen, America) and quantified by determination at OD_260_. RNA was extracted according to the manufacturer’s protocol, resuspended in 50 µL RNase-free water, and stored at −80°C. Quantification was done using Eppendorf Biophotometer (Hamburg, Germany). RNA was routinely treated with 100 U RNase-free DNaseI (promega) to avoid any DNA contamination. The purified total RNA (2 µg) was then reverse transcribed. Reverse transcription was performed with oligo (dT) 18 primer using First Strand cDNA synthesis kit (Toyobo, Japan) according to the manufacturer’s instructions. The resultant cDNA was then diluted 20 folds and kept at −20°C.

### Quantitative Real-Time PCR (Q-PCR)

All the primers used in Q-PCR were listed in Table 1. The Gene-specific primers were designed on line (http://www.idtdna.com/Scitools/Applications/Primerquest/Default.aspx) based on the gene sequences of *Rattus norvegicus* present on the NCBI homepage (http://www.ncbi.nlm.nih.gov). The specification of each pair of primers was confirmed by randomly sequencing six clones, and further confirmed by the melting curve analysis using Q-PCR. The amplification efficiency of each pair of primers was tested by constructing corresponding plasmid and only primers with similar amplification efficiency were used in this experiment.

**Table 1:** Real-time PCR primers used in this experiment.

Target gene	Primer Sequence (5'-3')		Size(bp)
	Forward	Reverse	
β-actin	CGTTGACATCCGTAAAGACCTC	TAGGAGCCAGGGCAGTAATCT	110
β-tubulin	GGAGCTGTTCAAGCGC	AGGTCATTCATGTTGCTCTC	129
vimentin	GGACCTGCTCAATGTAAAGATG	GGTGTCAACCAGAGGAAGTG	157
ezrin	GCTCCTGACTTCGTGTTCTAC	AGTTTCTCTCCTCTTCTTCTCG	207
radixin	ATGTGAGAGCCGTTCTTGTG	CAACTGTCTGAGTGCTTTGG	117
moesin	GGACCAGAAGAAGACTCAGG	CATTCCTCAGCCTCACTCTC	111
stathmin	GAGGAGAACAACAACTTCAGC	TCGGAACAACTTAGTCAGCC	199
COX I	TGAGCAGGAATAGTAGGGACA	GAGTAGAAATGATGGAGGAAGC	261
COX II	CTTATTCTTATCGCCCTCCC	GCTTCAGTATCATTGGTGACC	105
COX III	CAAGCCCTACTAATCACCATTC	GAAATCCCGTTGCTATGAAG	130
ND1	GCCACATCAAGTCTTTCAGTC	ATTTGTAGGGAGAAGGAGCC	173
ND3	CGACCTAGAAATCGCCTTAC	CATTCGTAGCTTAGGCCAAG	123
ND6	GTTAGTGGATGTATTGGGTGC	CCTCAGTAGCCATAGCAGTTG	130
ATPase6	GCAACCGACTACACTCATTTC	GGGATGGCTATGCTTAGGTC	205
ATPase8	TCATCTCCTCAATAGCCACAC	TGATTCTCAAGGGTTATTCGT	122
GAPDH	CGCTAACATCAAATGGGGTG	TTGCTGACAATCTTGAGGGAG	201

The housekeeping gene Glyceraldehyde 3-phosphate dehydrogenase (GAPDH) was analyzed in samples, and the level was stable in the present experiments; therefore GAPDH was used as the endogenous assay control. SYBR Green Q-PCR kit (TOYOBO, Japan) was used as the fluorescent dye for Q-PCR on a Chromo4 Real-Time Detection System (MJ Research, Cambridge, MA, USA). The reactions were performed in a 20 µL volume mix containing 10 µL SYBR Green I mixture, 1 µL primers, 1 µL cDNA, and 1 µL sterile, distilled- deionized water. Cycling conditions were as follows: 3 min at 95°C, 40 cycles of 15 s at 95°C, 20 s at 58°C, and 20 s at 72°C. Melting curve analysis of amplification products was performed at the end of each PCR reaction to confirm that a single PCR product was detected. Each sample was run in three tubes, and PCR reactions without the addition of the template used as blanks. After completion of the PCR amplification, data were analyzed with the Option Monitor software 2.03 version (MJ Research, Cambridge, MA, USA). The relative expression levels of the genes were calculated using the formula 2^−△△Ct^ method [44].

### Statistical Analysis

All results are expressed as means±SD and were subjected to one-way analysis of variance (ANOVA) and Duncan’s multiple comparison tests using Statistica software package (Version 6.0, Statsoft Inc.). Statistical differences were determined at the *p*<0.05 or *p*<0.01 levels for all analyses and indicated with * and **, respectively.

## Results

### Testis Index

**Figure 1:**
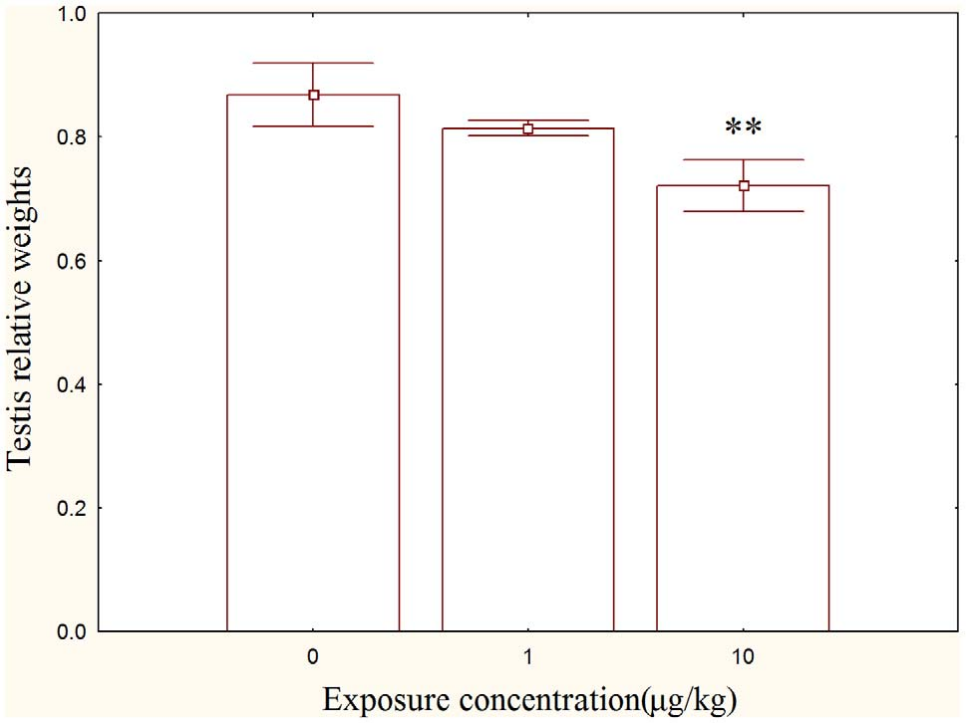
Effect of MC-LR on testis index rats in comparison to control rats.

No significant differences in testis index were observed between control and 1 µg/kg group (Fig. 1), but the testis index in the 10 µg/kg group was significantly decreased compared with the control (*p*<0.01).

### Light Microscope Observation

**Figure 2:**
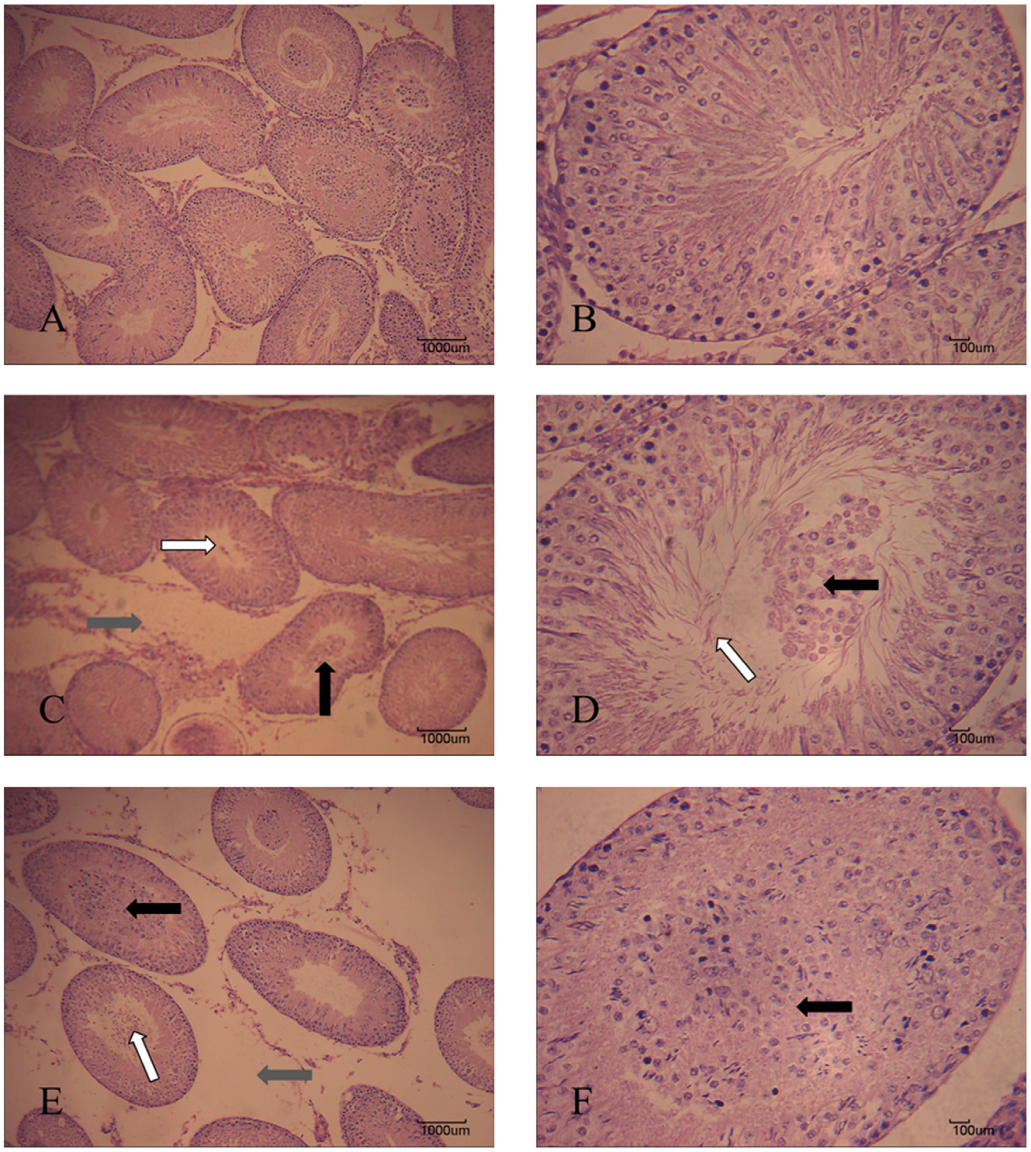
Testis microstructures of rats exposed to MC-LR by intraperitoneal injection at a dose of 1 or 10 µg/kg/day for 50 d (stained with H&E). (A) Control group (100×). (B) Control group (400×). (C) The 1 µg/kg/day group (100×), showing the enlarged spaces between the seminiferous tubules (gray arrow), enlargement of the lumen of the seminiferous tubules (white arrow), and blockage in seminiferous tubules (black arrow). (D) The 1µg/kg/day group (400×), showing the enlargement of the lumen of the seminiferous tubules (white arrow) and blockage in seminiferous tubules (black arrow). (E) The 10 µg/kg/day group, showing the enlarged spaces between the seminiferous tubules (gray arrow), enlargement of the lumen of the seminiferous tubules (white arrow), and blockage in seminiferous tubules (black arrow) (100×). (F) The 10 µg/kg/day group (400×), showing the blockage in seminiferous tubules (black arrow). Bar = 1000 µm (A, C, E) or 100 µm (B, D, F).

Morphological observation of testes was shown as Figure 2. In control, we observed a regular arrangement of spermatogenic epithelium, normal androgonial and spermatogenic cells in seminiferous tubules (Fig. 2 A and B). Enlarged spaces between the seminiferous tubules and the enlargement of the lumen of the seminiferous tubules were observed in 1 µg/kg group (Fig. 2 C and D). In 10 µg/kg group, more widened spaces between the seminiferous tubules were recorded (Fig. 2 E). In addition, the lumen of the seminiferous tubules was larger than in the control group or the low dose group (Fig. 2 F). Blockage in seminiferous tubules was observed in high dose group (Fig. 2 F).

### Electron Microscope Observations

**Figure 3:**
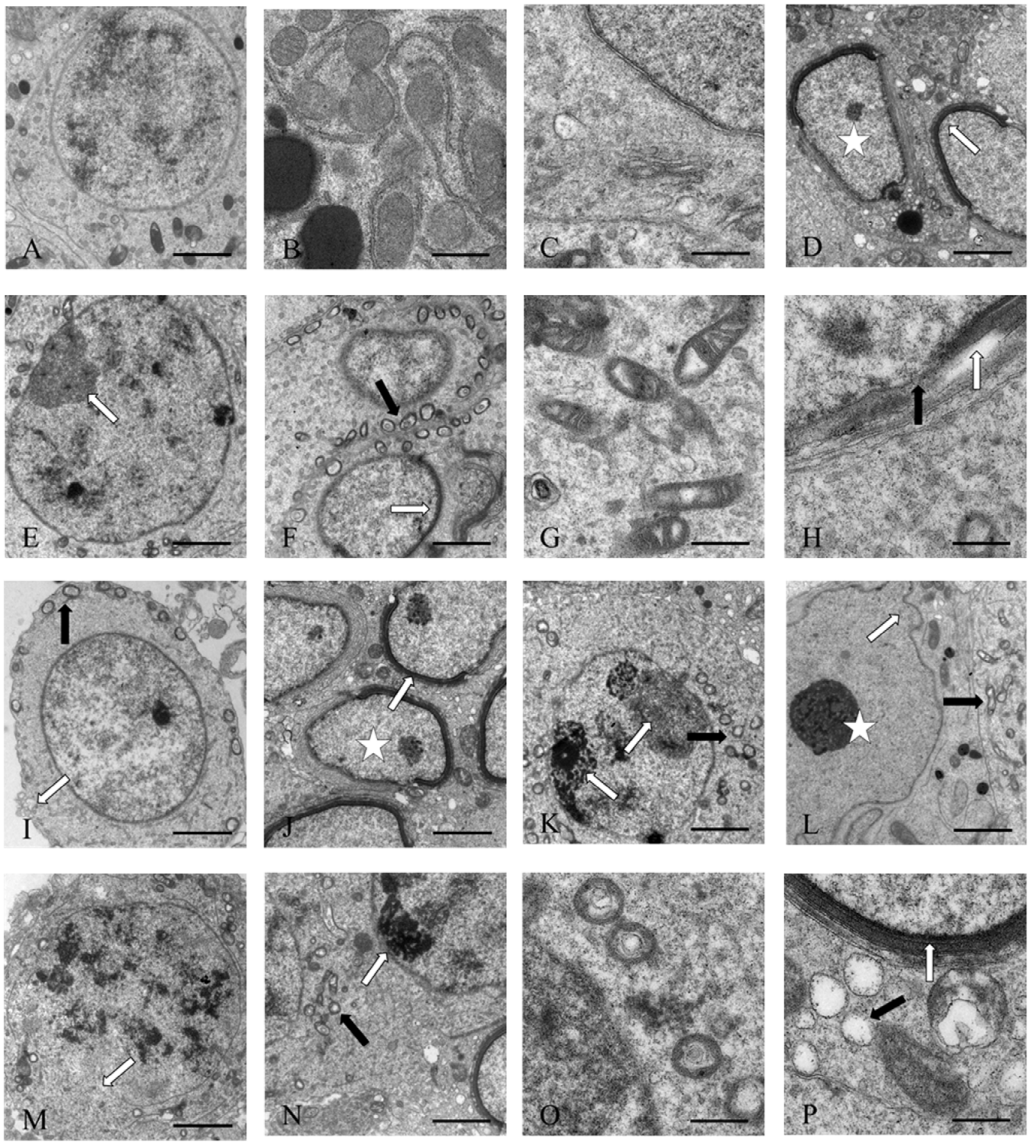
Toxic effect on testis ultrastructures of male rats treated with MC-LR. (A) showing spermatogonia of control rat, 2500×. (B) showing the normal mitochondria, 10000×. (C) showing the normal nucleus, 10000×. (D) showing the shrank cell (asterisk), margination of chromatin (arrow), 2500×. (E) showing the condensation of chromatin (arrow), 2500×. (F) showing the swollen mitochondria (black arrow), margination of chromatin (white arrow), 2500×. (G) showing the swollen mitochondria, 10000×. (H) showing the dissolved nucleus membrane (black arrow) and margination of chromatin (white arrow), 10000×. (I) showing the blebbing of spermatogonia (white arrow), swollen mitochondria (black arrow), 2500×. (J) showing the shrank cell (asterisk), margination of chromatin (arrow), 2500×. (K) showing the condensation of chromatin (white arrow), swollen mitochondria (black arrow), 2500×. (L) showing the nuclear shape alteration (white arrow), condensation of chromatin (asterisk), swollen mitochondria (black arrow), 2500×. (M) showing the dissolved nucleus membrane, 2500×. (N) showing the swollen mitochondria (black arrow), condensation of chromatin (white arrow), 2500×, (O) showing the swollen mitochondria, 10000×. (P) showing the swollen mitochondria (black arrow), margination of chromatin (white arrow), 10000×.

In the control group (Fig. 3 A, B and C), spermatogonia had a clear and complete cell structure with obvious cell membrane and nucleolus, with no signs of any pathological change. In the low dose group (Fig. 3 D, E, F, G and H), spermatogonia showed evident apoptotic characters, with the observation of shrank cell, condensation and margination of chromatin, leading to the formation of a ring at the inner side of the nuclear envelope. Swollen mitochondria were also observed. In the high dose group (Fig. 3 I, J, K, L, M, N, O and P), the spermatogonia began to bleb, cytoplasmic shrinkage was more evident, and nuclear changes became more pronounced, the numerous apoptotic nuclei such as chromatin condensation, margination, the formation of a ring at the inner side of the nuclear envelope, and cells that lacked part of their nuclear envelope, nuclear shape alteration were also observed. Most mitochondria were seriously swelling and the contents were missing in testis of rats exposed to MC.

### Mitochondrial Swelling and DNA Damage

**Figure 4:**
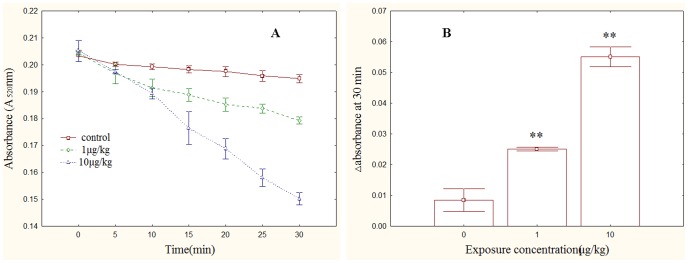
MC-Expose induced swelling of male rat testis mitochondria. (A) MPT was measured as a decrease in absorbance at a 520 nm wavelength (A_520_) in isolated rat testis mitochondria. (B) A total change in A_520_ over 30 min of mitochondrial swelling, **, p<0.01 versus control.

**Figure 5:**
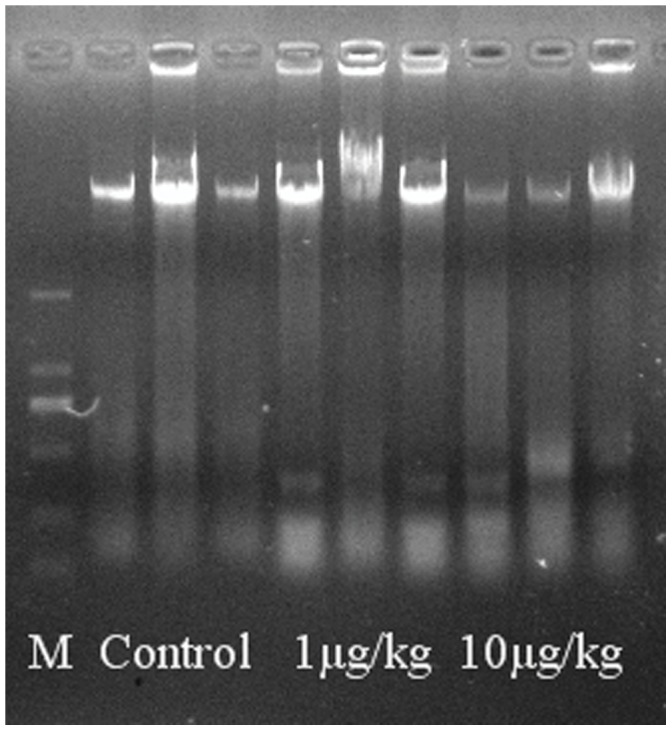
MC-induced mitochondrial DNA damage of rat testis. Electrophoresis was carried out for 0.5 h in 1% agarose gel. DL 2000 DNA marker was served as molecular size standard. MtDNA were stained with GelGreen.

Mitochondrial Swelling was examined by the absorbance of A_520_. Time dependent effect changes of mitochondrial swelling were observed (Fig. 4. A). Significantly increased absorbance of A_520_ was determined in MC-LR treatments (*p*<0.01), with a dose dependent effect (Fig. 4. B). Mitochondrial DNA electrophoresis result indicated that some DNA fragmentation was observed in both MC-LR treatment groups (Fig. 5).

### Tissue Hormone Analysis and ROS Determination

Tissue hormone analysis and ROS determination were shown as Table 2. In the 10 µg/kg group, the FSH level was significantly higher compared with the control group (*p*<0.05). Compared with the control, significantly increased LH levels were observed both in low dose group (*p*<0.05) and high dose group (*p*<0.01). The testosterone levels in MC-LR treatment groups significantly decreased by comparison with that of the control (*p*<0.01). Compared with the control, there was not significant difference of ROS production in 1 µg/kg group. However, the ROS production was significantly increased in the 10 µg/kg group compared with the control (*p*<0.05).

**Table 2:** Concentration of hormone and reactive oxygen species (ROS) levels in rat testes exposed to MC-LR.

	Control	1µg/kg	10 µg/kg
Testosterone (100 ng/g)	8.30±0.79	6.91±0.87^**^	4.79±0.53^**^
FSH (10 mIU/g)	1.77±0.10	1.84±0.09	1.93±0.13^*^
LH (100 mIU/g)	2.13±0.10	2.31±0.07^*^	2.43±0.21^**^
ROS (U/mg protein)	55.13±7.29	52.23±4.20	64.16±11.27^*^

### Transcription of Cytoskeletal Genes

**Figure 6:**
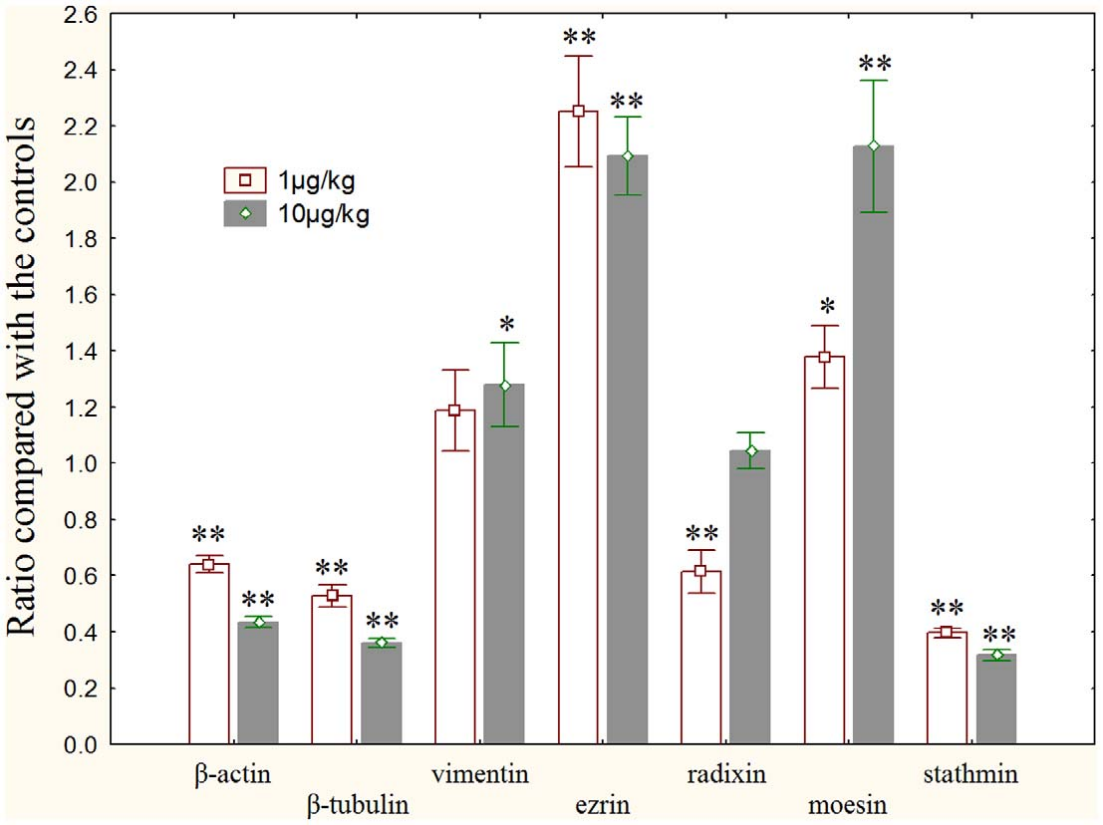
Transcriptional changes of cytoskeletal genes in the testis of male rats exposure to MC-LR compared with controls. Quantitative real-time PCR was used to test the expression levels of cytoskeletal genes. GAPDH was used as an internal control. *, *p*<0.05 versus control, **, *p*<0.01 versus control.

The transcriptional changes of cytoskeletal genes were shown as Fig. 6. Compared with the control, the transcriptional levels of β-actin and β-tubulin were significantly decreased in the MC-LR treatment groups (*p*<0.01). No significant alteration of vimentin transcription was detected in the low dose group, however, significant increase was observed in high dose group (*p*<0.05). The transcription of ezrin in testes tissue was obviously augmented in both MC-LR treatment groups (*p*<0.01). Radixin mRNA level was significantly lower in the low dose group (*p*<0.01), and no prominent change was found in the high dose group. A dose-dependent increase of moesin transcription was observed in 1µg/kg group (*p*<0.05) and 10µg/kg group (*p*<0.01). The transcription level of stathmin was significantly decreased in both MC-LR treatments (*p*<0.01).

### Transcription of Mitochondrial Genes

**Figure 7:**
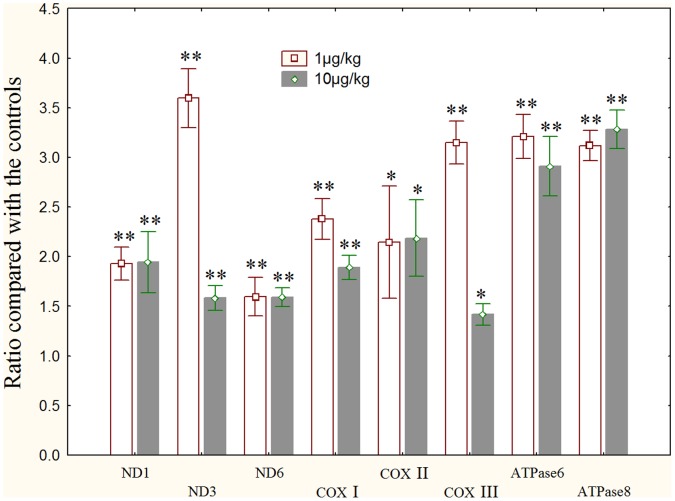
Transcriptional changes of mitochondrial genes in the testis of male rats exposure to MC-LR compared with controls. Quantitative real-time PCR was used to test the expression levels of mitochondrial genes. GAPDH was used as an internal control. *, *p*<0.05 versus control, **, *p*<0.01 versus control.

All of the 8 mitochondrial genes, including COXs(-I, -II, -III), NDs (-1, -3, -6) and ATPases (-6, -8) were significantly elevated in the two MC-LR treatment groups (Fig. 7).

## Discussion

The exposure to many environmental toxicants leads directly to a notable decline in fertility of animals and humans [13]. Several recent studies have demonstrated the reproductive toxicity of MCs to mammals [7], [8], [12], [13], [45], [46]. Most of these studies conducted their investigation using acute exposure way. In fact, a chronic exposure should be more proper to determine the impairment of reproductive system. In the present study, after a 50 d exposure of MC-LR, a significant decrease in testes index was observed in high dose group, and this decline was also consistent with the results of testicular atrophy in morphological observation. Similar reproductive system disruption was also reported by several other studies [7], [12], [13]. It can be deduced that MCs can pass through the blood-testis barrier (BTB) and cause morphological damage of testes. The present ultrastructural observation indicated some typical apoptotic features, including cell membrane blebbing, cytoplasmic shrinkage, swollen mitochondria, and deformation of the nucleus. Majsterek et al. [47] reported that the morphological changes in mitochondria are the primary effect induced by MC-LR leading to cell death. Several studies showed MC-induced cell junction and plasma membrane injury [8], [10], [26]. Cytoskeleton disruption may affect membrane, intercellular junction, chromatin structures, and other cell morphology [21], [26], [48].

Testosterone, a kind of steroid hormone, plays an important role in reproductive tract development, spermatogenesis, and erectile function and may be disrupted by diverse environmental toxicants [7]. In the present study, MC-LR exposure significantly decreased testis testosterone level while increased FSH and LH level; as the secretion of FSH and LH is regulated by testosterone in a negative feedback manner [7], [13]. So the hormone changes in the present study encourage us to affirm that reproductive function obviously ebbed in testis of rats treated with MC-LR. MC-LR changed the serum hormones by directly damaging Leydig cells and affected testosterone synthesis [7]. Cytoskeletal polymers such as microfilaments and microtubules have been implicated in regulating steroidogenesis [49]. Considering the disturbed cytoskeletal gene transcription and damaged cellular structure in our study, we believe that the cytoskeleton disruptions are supposed to impair the testosterone synthesis ability.

Cytoskeleton disruption is one of the first striking cytotoxicity following MC-LR exposure [27]. The cytoskeleton, a basic structural element of all cell types, plays key roles in the maintenance of cell architecture, adhesion, migration, differentiation, division and organelle transport. MCs can lead to rapid reorganization of all three major cytoskeletal components, microfilaments, microtubules and intermediate filaments [20]. The disruption of the intermediate filaments could be attributed to MC-LR-induced hyperphosphorylation of keratins 8 and 18 *in vivo* [25] and *in vitro* [23], [24]. MC-LR leads to the reorganization of cytoskeletal architectures in PC12 cells and hyperphosphorylation of tau and HSP27, which may be caused by direct PP2A inhibition and indirect p38 MAPK activation [50]. Gácsi et al. [21] found that MC-LR leads to the shortening and loss of actin filaments and depolymerization of microtubules on Chinese hamster ovary (CHO-K1) cells. MC-LR treatment induced microtubule disorganization or disruption was also observed in interphase cells of primary and lateral root meristems, and in the elongation and differentiating root tissues of common reed (*Phragmites australis*) [22]. Fu et al. [51] showed that some cells lose microtubules after MCs treatment except the reorganization and aggregation of microtubules. Our previous research [52] also determined transcriptional changes of some cytoskeletal genes in liver, kidney and spleen in rats treated with MCs. However, such cytoskeletal genes have not been examined in gonads yet. The present study verified that MC-LR remarkably disrupt the transcriptional balance of some cytoskeletal genes. So we assume that MC-LR exposure can significantly affect cytoskeleton organization in rat testis due to the altered expressions of MFs, MTs and IFs, thus leading to the morphological changes, and exert prominent toxicity to reproductive system.

Mitochondria are known to be the vulnerable target of MCs [35]. The principal function of mitochondria is to produce energy, which is achieved by the electron transport chain (ETC) and oxidative phosphorylation (OXPHOS), which consists of five multi-protein complexes [53]. Mitochondrial ETC is considered as a major intracellular source of ROS [54], mainly at the level of the complex I and III [55]. The 8 mitochondrial genes in the present study are important components of ETC and OXPHOS systems, including NADH dehydrogenase (NDs, -1, -3, -6), cytochrome c oxidase (COX, -I, -II, -III) and ATP synthase (ATPS, -6, -8). MC-LR led to the uncoupling of mitochondrial electron transport and production of ROS [47], [56]. La-Salete et al [38] found a significant effect of MC-LR on mitochondrial OXPHOS system and respiratory chain of isolated mitochondria from kidney of rat. Our previous study indicated that the toxic effect of MCs was through influencing the mitochondrial ETC and phosphorylation system, and the decrease of NADH dehydrogenase activity was detected in rabbit liver [33]. Several proteomic studies revealed the altered abundance of NADH dehydrogenase Fe-S protein 8 in mouse liver [57], NADH dehydrogenase [ubiquinone] 1 alpha subcomplex subunit 10 (NDUFA10) in zebrafish brain [58] and NADH-ubiquinone oxidoreductase 75 kDa subunit in zebrafish testis [10]. Cytochrome c oxidase (COX) is the terminal enzyme of mitochondrial ETC. Changes in the activity of cytochrome c oxidase caused by MC can be used as a proxy related to cell response *in vivo* [47]. MC-LR has been shown to be able to bind the beta subunit of ATP synthase, which could be associated with the suggested apoptosis-inducing potential of MCs [59]. MC-LR induced an inhibition of ATP synthase activities in rat kidney mitochondria [38]. In our previous proteomic study, significantly increased ATP synthase, H1 transporting mitochondrial F1 complex and b subunit (ATP5B, increase) were identified in zebrafish embryos with MC-LR treatment [18]. Interestingly, in both MC-LR treatment groups, transcriptional levels of 8 genes all were elevated in testes of rats. We think that the raised transcriptional level of mitochondrial genes is a kind of compensatory mechanism after mitochondria structure being destroyed in testis of rat. This compensatory response also has been reported in zebrafish embryo in our previous studies [18]. In fact, this is the first study investigating mitochondrial genes transcriptional changes of gonads.

The genotoxicity of MC-LR is mediated by its induction of ROS formation, which causes formation of DNA strand breaks and mutagenic oxidative DNA lesions [60], [61]. Lankoff et al. [62] believe that MCLR-induced DNA damage may be related to the early stages of apoptosis due to cytotoxicity. ROS formation is intrinsically related with mitochondria metabolism and can cause cell death by necrosis or apoptosis and to genotoxicity [2]. MtDNA represents an important target for in MC-induced oxidative damage, and if not repaired, can lead to mitochondria dysregulation and cell death [63]. In the present study, we think that testis mtDNA impairment should be caused by oxidative damage due to excessive ROS formation. Therefore, our results of gene transcription, swelling measurement, ROS production and DNA fragments convinced the distinct damage of mitochondria in testes of rats caused by MC-LR exposure.

In the present study, we believe that the cytoskeleton disruption and mitochondria dysfunction interact with each other through ROS formation and jointly result in impairment of reproductive system. On one hand, the impaired mitochondrial OXPHOS system may increase ROS formation, especially superoxide radical, which plays a crucial role in the disruption of cytoskeleton organization [28], [64]. ROS can exert direct oxidative injuries on actin [65] and result in depletion of protein sulfhydryl (-SH) groups [66]. Moreover, it was suggested that oxidative stress led to the elevation of intracellular Ca^2+^, which may also cause the cytoskeleton alterations [64], [67]. On the other hand, cytoskeletal organization is crucial for normal mitochondrial morphology, motility and distribution and function. Cytoskeleton is involved in the metabolic regulation of mitochondrial respiration and energy fluxes [68]. Cytoskeleton acts as a regulator of oxidative stress in the cell [69]. Recent evidence from diverse eukaryotic systems suggests that the actin cytoskeleton plays a key role in regulating apoptosis via interactions with the mitochondria; meanwhile, this interaction also appears to have a significant impact on the management of oxidative stress [69], [70].

In conclusion, our *in vivo* study confirms that MC-LR can exert a generally chronic toxicity to male rat reproductive system through influencing the cytoskeleton and mitochondria. Transcriptional alteration of cytoskeletal genes has a high probability with cytoskeleton structural disruption and cell death. Further researches are needed to reveal mechanisms of the cytoskeleton damage. Cytoskeleton disruption could interact with mitochondria dysfunction, ultimately leading to disruption of cellular structure and metabolism. MC-LR injures mitochondrial respiratory chain and oxidative phosphorylation system, which might be responsible for promoted ROS formation and oxidative stress and leads to cytoskeletal disruption and hormone homeostasis. Chen et al. [1] have identified high levels of MCs in serum of fisherman in China. So MCs have been a great risk to human reproduction function. More future works are needed to elucidate the mechanisms of reproductive toxicity caused by microcystins exposure.
